# Contemporary validation of a SAPS 3 customized version in patients admitted to Brazilian and Uruguayan intensive care units: a multicenter cohort study

**DOI:** 10.62675/2965-2774.20260334

**Published:** 2026-03-16

**Authors:** Marcio Soares, Lunna Perdigão Borges, Gastón Burghi, Pedro Kurtz, José Raimundo Araújo de Azevedo, Carlos Eduardo Brandão, Aloysio Saulo Breves Beiler, Niklas Soderberg Campos, Liane Oliveira Cavalcante, Mario Diego Teles Correia, Victor de Souza Cravo, Pedro Henrique Barbosa D’Almeida, Flávio Geraldo Rezende de Freitas, Thais de Almeida Machado, Marcelo de Oliveira Maia, Edson Silva Marques, Gloria Adriana Rocha Martins, Ulisses de Oliveira Melo, Laura Herranz Prinz, Silvia Regina Ramos, Thiago Gomes Romano, Marcos Soares Tavares, Suzana Margareth Lobo, Jorge Ibrain Figueira Salluh, Ederlon Rezende

**Affiliations:** 1 Instituto D’Or de Pesquisa e Ensino Department of Critical Care and Postgraduate Program Rio de Janeiro RJ Brazil Department of Critical Care and Postgraduate Program, Instituto D’Or de Pesquisa e Ensino - Rio de Janeiro (RJ), Brazil.; 2 Epimed Solutions Department of Data Science Rio de Janeiro RJ Brazil Department of Data Science, Epimed Solutions - Rio de Janeiro (RJ), Brazil.; 3 Intensive Care Unit Hospital Maciel Montevideo Uruguay Intensive Care Unit, Hospital Maciel - Montevideo, Uruguay.; 4 Intensive Care Unit Hospital São Domingos São Luís MA Brazil Intensive Care Unit, Hospital São Domingos - São Luís (MA), Brazil.; 5 Amil Hospital Network Critical Care Department São Paulo Brazil Critical Care Department, Amil Hospital Network - São Paulo, Brazil.; 6 Intensive Care Unit Hospital Copa D’Or Rio de Janeiro RJ Brazil Intensive Care Unit, Hospital Copa D’Or - Rio de Janeiro (RJ), Brazil.; 7 Intensive Care Unit Hospital Municipal Moysés Deutsch São Paulo SP Brazil Intensive Care Unit, Hospital Municipal Moysés Deutsch - São Paulo (SP), Brazil.; 8 Intensive Care Unit Hospital Delphina Rinaldi Abdel Aziz Manaus AM Brazil Intensive Care Unit, Hospital Delphina Rinaldi Abdel Aziz - Manaus (AM), Brazil.; 9 Intensive Care Unit Real Hospital Português Recife PE Brazil Intensive Care Unit, Real Hospital Português - Recife (PE), Brazil.; 10 Intensive Care Unit Americas Medical Center Rio de Janeiro RJ Brazil Intensive Care Unit, Americas Medical Center - Rio de Janeiro (RJ), Brazil.; 11 Intensive Care Unit Hospital Pasteur Rio de Janeiro RJ Brazil Intensive Care Unit, Hospital Pasteur - Rio de Janeiro (RJ), Brazil.; 12 Intensive Care Unit Hospital SEPACO São Paulo SP Brazil Intensive Care Unit, Hospital SEPACO - São Paulo (SP), Brazil.; 13 Intensive Care Unit Hospital Anchieta Taguatinga DF Brazil Intensive Care Unit, Hospital Anchieta - Taguatinga (DF), Brazil.; 14 Intensive Care Unit Hospital Cardiopulmonar Salvador BA Brazil Intensive Care Unit, Hospital Cardiopulmonar - Salvador (BA), Brazil.; 15 Intensive Care Unit Hospital Estadual Alberto Torres São Gonçalo RJ Brazil Intensive Care Unit, Hospital Estadual Alberto Torres - São Gonçalo (RJ), Brazil.; 16 Intensive Care Unit Hospital Quinta D’Or Rio de Janeiro RJ Brazil Intensive Care Unit, Hospital Quinta D’Or - Rio de Janeiro (RJ), Brazil.; 17 Intensive Care Unit Hospital Assunção São Bernardo do Campo SP Brazil Intensive Care Unit, Hospital Assunção - São Bernardo do Campo (SP), Brazil.; 18 Intensive Care Unit Hospital Vila Nova Star São Paulo SP Brazil Intensive Care Unit, Hospital Vila Nova Star - São Paulo (SP), Brazil.; 19 Intensive Care Unit Hospital Nove de Julho São Paulo SP Brazil Intensive Care Unit, Hospital Nove de Julho - São Paulo (SP), Brazil.; 20 Hospital de Base Department of Critical Care São José do Rio Preto SP Brazil Department of Critical Care, Hospital de Base - São José do Rio Preto (SP), Brazil.; 21 Hospital do Servidor Público Estadual ¨Francisco Morato de Oliveira" Department of Critical Care São Paulo SP Brazil Department of Critical Care, Hospital do Servidor Público Estadual ¨Francisco Morato de Oliveira" - São Paulo (SP), Brazil.

**Keywords:** Calibration, Hospital mortality, Length of stay, Simplified Acute Physiology Score, Benchmarking, Intensive care units, Brazil, Uruguay

## Abstract

**Objective:**

To compare the performance of the standard equation (SAPS 3-SE) and a customized version (SAPS 3-Custom) of the Simplified Acute Physiology Score 3 in a contemporary cohort of Brazilian and Uruguayan intensive care unit patients.

**Methods:**

We conducted a retrospective cohort study of 262,198 adults admitted to 177 intensive care units between 2022 and 2023. Discrimination was assessed using the area under the Receiver Operating Characteristic curve (AUROC), and calibration by comparing predicted and observed mortality in calibration curves.

**Results:**

Of patients 70% were medical, and 21% were scheduled for surgery; mean SAPS 3 was 46.6 ± 16.0. Median intensive care unit and hospital stays were 3 (1 - 5) and 8 (4 - 16) days, respectively. Intensive care unit mortality was 10.6% and hospital mortality was 16.4%. Predicted mortality was 19.0% for SAPS 3-SE and 16.6% for SAPS 3-Custom. Both models had excellent discrimination (AUROC = 0.841). SAPS 3-SE overestimated mortality across all risk deciles, whereas SAPS 3-Custom achieved uniform agreement between predicted and observed values. Standardized mortality rates were 0.86 (95%CI 0.85 - 0.87) for SAPS 3-SE and 0.98 (0.98 - 0.99) for SAPS 3-Custom; standardized resource use rates were 0.90 (0.90 - 0.91) and 0.98 (0.97 - 0.98), respectively. At the intensive care unit level, SAPS 3-Custom produced standardized mortality rates (0.95 [0.77 - 1.17]) and standardized resource use rates (0.97 [0.82 - 1.23]) distributions centered around 1.0, unlike SAPS 3-SE, which yielded lower values. Findings were consistent for medical and surgical subgroups.

**Conclusion:**

In this large, contemporary cohort, SAPS 3-Custom demonstrated superior calibration and accuracy over SAPS 3-SE, supporting its use for performance evaluation and benchmarking in intensive care units in Brazil and Uruguay.

## INTRODUCTION

Severity of illness scores (SOIS) are routinely used to evaluate and benchmark intensive care unit (ICU) performance and efficiency, and to assess temporal, severity-adjusted trends in mortality.^(1-3)^ The Simplified Acute Physiology Score (SAPS) 3 was published in 2005 and developed in a database of 16,784 patients admitted to 303 ICUs from 35 countries, including Brazil.^(4)^ Since 2009, the *Associação de Medicina Intensiva Brasileira* (AMIB) has defined the SAPS 3 score as the recommended SOIS to assess performance and to benchmark Brazilian ICUs.^(5)^ Different multicenter studies have supported this decision.^(6-8)^ The last extensive multicenter validation study was published in 2017 using data from 48,818 patients admitted to 70 ICUs in 50 hospitals during 2013.^(6)^ In that study, the SAPS 3 standard equation (SAPS 3-SE) had both good discrimination and calibration, while the customized equation for Central and South American countries overestimated mortality.^(6)^ However, the performance of SOIS is expected to deteriorate over time, particularly in terms of calibration, thus requiring periodical reassessments.^(9,10)^ The SAPS 3 also allows the evaluation of ICU efficiency using the standardized resource use rate (SRU).^(11)^ Nevertheless, the parameters and metrics to estimate the SRU were reported in 2007 by Rothen et al. using the SAPS 3 original dataset, and, to our knowledge, they have not been revalidated yet.^(11)^ Moreover, a few years ago, the critical care setting was seriously challenged and affected worldwide by the coronavirus disease 2019 (COVID-19) pandemic, with significant changes in patients’ clinical management and how the ICUs are organized and managed,^(12)^ which raises concerns about whether the standard SAPS 3 parameters remain accurate in current practice.

The *UTIs Brasileiras* (Brazilian ICUs) is a national critical care registry that encompasses approximately half of adult ICUs in Brazil.^(13)^ The aforementioned concerns led to an evaluation of SAPS 3 performance in the *UTIs Brasileiras* database, including more than 1.3 million patients admitted to 1,239 ICUs in 562 hospitals during 2023 and 2024, which resulted in a "white paper" available at the registry website.^(13)^ This assessment found that although the model's discrimination remained very good, the calibration was poor, resulting in overestimation of hospital mortality. In addition, the parameters used to estimate the SRU were inappropriate, leading also to overestimation of observed resource use. Therefore, the researchers performed a first-level recalibration of the SAPS 3 equation (SAPS 3-Custom) and updated the average number of expected ICU days to produce a survivor using the same stratification according to the severity of illness originally proposed by Rothen et al.^(11)^ The customized equation and the new parameters resulted in more accurate estimates of both standardized mortality rates (SMR) and SRU in that cohort. However, to our knowledge, this customized version of SAPS 3 has not yet been externally validated. Therefore, the present study aimed to compare the performance of the standard equation (SAPS 3-SE) and a customized version (SAPS 3-Custom) of SAPS 3 in a contemporary cohort of Brazilian and Uruguayan ICU patients.

## METHODS

### Study design and setting

We performed a retrospective analysis of prospectively collected data from patients admitted to 177 ICUs (169 in Brazil and 8 in Uruguay) across 99 hospitals (91 in Brazil and 8 in Uruguay) between January 1^st^, 2022, and December 31^st^, 2023. All participating centers and investigators are listed in the Appendix 1. The Brazilian National Ethics Committee (Brazil CAAE: 19687113.8.1001.5249) and the Ethics Committee of the Hospital Maciel, Montevideo, Uruguay (protocol number 20/2017) approved the study and waived the need for informed consent.

### Selection of participants, data collection and definitions

The included ICUs participate in the Organizational Characteristics in Critical Care (ORCHESTRA) and in the Brazilian Research in Intensive Care (BRICNet) networks.^(14,15)^ Cardiac and burn ICUs, as well as those with < 8 beds, were not included. We excluded patients with less than 16 years, readmitted to the ICU during the same hospitalization (i.e., only the first ICU admission was considered), with ICU LOS < 6 hours or hospital LOS > 90 days, potential organ donors, and brain dead at ICU admission. In addition, we excluded patients who were missing core data (admission source, primary admission diagnosis, ICU, and hospital outcomes) or had outlier or non-plausible data values.

De-identified patient data routinely collected by trained personnel were retrieved from the Epimed Monitor System (Epimed Solutions®, Rio de Janeiro, Brazil), a cloud-based registry for ICU quality improvement and benchmarking purposes.^(16)^ Data collection in the participant ICUs was a varying combination of integration with the hospital's electronic medical and/or administrative records (HER) and manual data entry, depending on the hospital's information technology infrastructure. In most ICUs, administrative (demographics, ICU, and hospital admission/discharge information) is integrated, and a dedicated case manager (usually nurses) is responsible for entering the remaining clinical and laboratory data for every consecutive patient into the database. Collected data included demographics, admission source, hospital length-of-stay (LOS) before ICU admission, primary ICU admission diagnosis, the Sequential Organ Failure Assessment (SOFA) score at admission,^(17)^ comorbidities based on Charlson Comorbidity Index (CCI),^(18)^ use of organ support at admission, ICU and hospital LOS, vital status at hospital discharge (dead or alive) and destination after hospital discharge. A full description of the database is provided elsewhere.^(16)^

### Outcomes

The primary outcome was all-cause in-hospital mortality at the patient level. The ICU LOS was the secondary outcome.

### Missing data

Following the recommendations for the SAPS 3 calculation, we input normal values for laboratory and physiological variables.^(4)^Table 1S (Supplementary Material) presents the frequencies of missing data for each SAPS 3 variable or component.

### Statistical analysis

We described ICU and patient characteristics using standard descriptive statistics and reported continuous variables as mean ± standard deviation or median (25% - 75% interquartile range [IQR]), as appropriate. We reported categorical variables as absolute numbers (frequency percentages).

We calculated the probability of death [e^logit^/(1+e^logit^)] using both logits of SAPS 3-SE {[-32.6659 + ln(SAPS 3 score + 20.5958) x 7.3068]}^(4)^ and of SAPS 3-Custom [-20.9447434 + ln(SAPS 3 score + 1) × 4.894223]}.^(5)^ Then, we evaluated models’ discrimination (i.e., the ability of each model to discriminate between patients who lived and those who died) by estimating the area under the Receiver Operating Characteristic curve (AUROC). We plotted calibration curves with respective 95% confidence interval (95%CI) to investigate the relationship between the observed and expected outcomes. For the calibration curves, patients were stratified into 10% risk deciles or into deciles with an equal number of patients. Hosmer-Lemeshow goodness-of-fit tests with C and H statistics were used to evaluate agreement between the observed and expected numbers of survivors and non-survivors across all strata of probability of death.^(19)^ We estimated both SMR and SRU rates with respective 95%CI to evaluate, respectively, clinical performance and efficiency in resource use. The SMR is the ratio of observed to predicted hospital mortality. The SRU estimates the average observed-to-expected ratio of resources (based on ICU LOS) used per surviving patient in a specific ICU adjusted for the SAPS 3 using the original parameters reported by Rothen et al.^(11)^ and the customized ones in the *UTIs Brasileiras* database^(5)^ (Table 2S - Supplementary Material). Zero expected day was assigned to non-survivors. We performed prespecified sensitivity analyses by admission type (medical *versus* surgical). We report a summary of SAPS 3-Custom model development in Supplementary Material.

We performed all statistical analyses using R (version 4.4.1)^(20)^ and followed the Strengthening the Reporting of Observational Studies in Epidemiology (STROBE) reporting guidelines^(21)^ in this article.

## RESULTS

### Characterization of the studied population and participating centers

**Figure 1:**
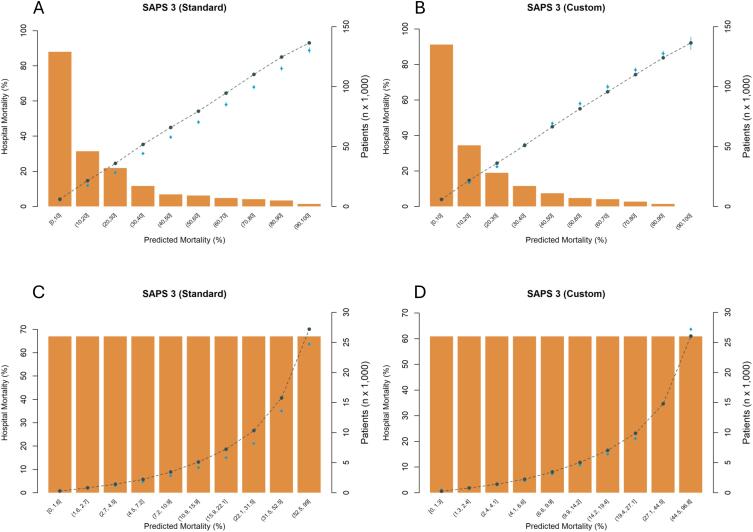
Calibration curves for the standard (SAPS 3-SE) and customized (SAPS 3-Custom) equations in all admissions (n = 262,198).

One hundred seventy-seven ICUs from 99 hospitals participated in the study. Of 298,124 admissions, 262,198 were deemed eligible and formed the study population. The study flowchart is shown in figure 1S (Supplementary Material). Table 3S (Supplementary Material) depicts the main ICU characteristics. Intensive care units were, in general, medical-surgical (76.8%) and located in private hospitals (64.4%).

The median number of patients per ICU was 1,208 (IQR 847 - 1,901). Table 1 summarizes the main patient characteristics and outcomes. Most admissions (70%) were due to medical complications, with sepsis, cardiovascular events, and neurological conditions being the most common. Scheduled surgeries accounted for 21% of admissions. At ICU admission, invasive mechanical ventilation (MV) was used in 14.5% of patients, vasopressors in 16.1%, and renal replacement therapy in 2.1%. The median LOS was 3 days (IQR 1 - 5) in the ICU and 8 days (IQR 4 - 16) in the hospital. ICU mortality was 10.6%, while hospital mortality was 16.4%.

**Table 1:** Main patient characteristics (n = 262,198)

Characteristics		
Age (years)		62.9 ± 19.2
Female sex		131,087 (50.0)
Charlson Comorbidity Index (points)		1.0 (0.0 - 2.0)
Hospital LOS before ICU admission (days)		0.0 (0.0 - 1.0)
SAPS 3 (points)		46.6 ± 16.0
SOFA score (points)		2.0 (0.0 - 4.0)
Admission source		
	Emergency room	131,035 (50.0)
	Operating room	63,332 (24.2)
	Ward/floor	27,808 (10.6)
	Transfer from another hospital	20,487 (7.8)
	Cardiovascular intervention room	8,190 (3.1)
	Other	11,346 (4.3)
Admission type/admission category		
	Scheduled surgery	54,986 (21.0)
	Emergency surgery	23,024 (8.8)
	Medical	184,188 (70.2)
	Sepsis	53,156 (20.3)
	Cardiovascular	42,158 (16.1)
	Neurological	30,733 (11.7)
	Renal/metabolic	11,809 (4.5)
	Respiratory	10,377 (4.0)
	Gastrointestinal	3,337 (1.3)
	Other	32,618 (12.5)
Invasive support ±1 hour from admission		
	Mechanical ventilation	38,559 (14.8)
	Vasopressors	42,184 (16.1)
	Renal replacement therapy	5,505 (2.1)
ICU LOS (days)		3.0 (1.0 - 5.0)
Hospital LOS (days)		8.0 (4.0 - 16.0)
ICU mortality		27,886 (10.6)
Hospital mortality		42,934 (16.4)
Destination after hospital discharge in surviving patients		
	Home	202,947 (77.7)
	Other hospital	7,240 (2.8)
	Hospice/nursing facility	1,529 (0.6)
	Unknown/other	6,663 (2.5)

LOS - length of stay; ICU - intensive care unit; SAPS - Simplified Acute Physiology Score; SOFA - Sequential Organ Failure Assessment. Results expressed as mean ± standard deviation, n (%), or median (interquartile range).

### Performance analysis of the SAPS 3-SE and SAPS 3-Custom

**Figure 2:**
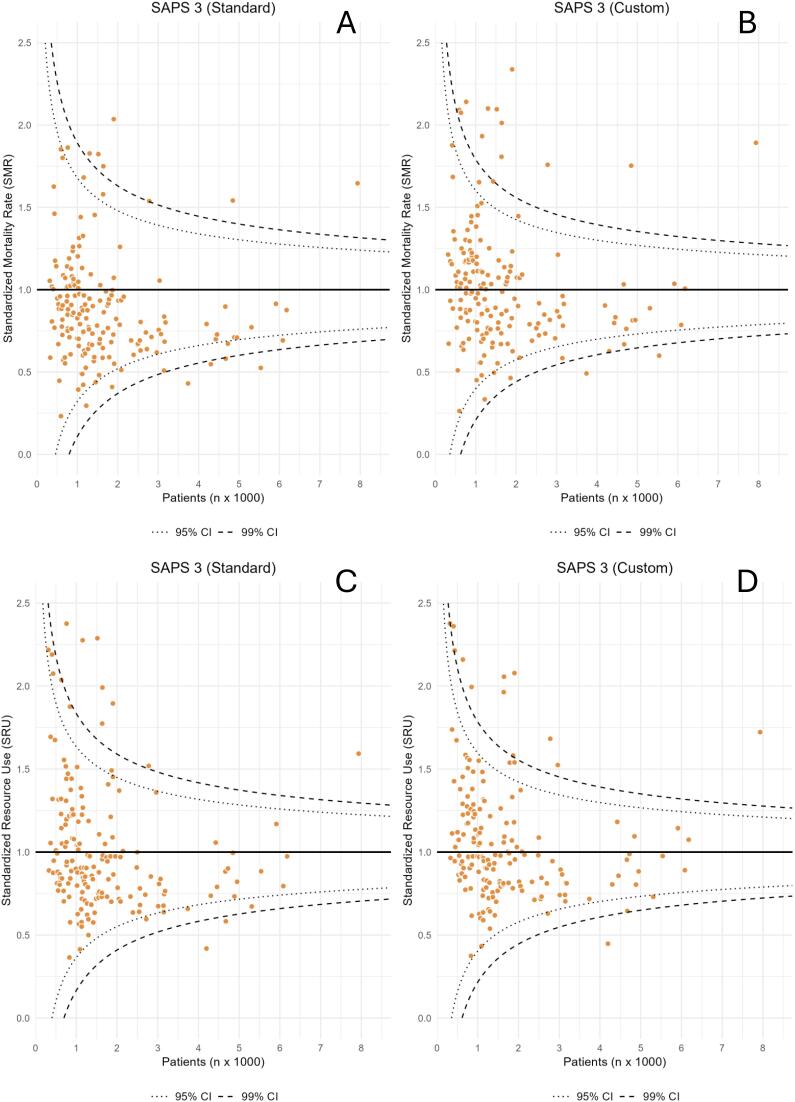
Funnel plot graphs of individual standardized mortality (panels A and B) and resource use (panels C and D) rates using the original (SAPS 3-SE) and SAPS 3-Custom equations in all intensive care units (n = 177).

The mean SAPS 3 score was 46.6 ± 16.0 points. Table 2 presents the performance analyses using both the standard and customized equations. For the overall patient cohort, discrimination was excellent [AUROC = 0.841 (95%CI 0.839 - 0.841)]. In the calibration analysis, the SAPS 3-SE showed poor fit, overestimating mortality across all risk ranges (Figures 1A and 1C) and yielding a global SMR of 0.86 (95%CI 0.85 - 0.87) (Table 2). In contrast, the SAPS 3-Custom produced mortality estimates closer to the observed values, with better calibration across all risk strata and an overall SMR of 0.98 (95%CI 0.98 - 0.99) (Figures 1B and 1D; Table 2). Efficiency analysis showed a similar pattern: the SAPS 3-Custom outperformed the SAPS 3-SE, with SRU = 0.98 (95%CI 0.97 - 0.98) *versus* SRU = 0.90 (95%CI 0.90 - 0.91), respectively. At the ICU level, funnel plot analyses demonstrated that the SAPS 3-Custom resulted in a more balanced distribution of SMR [median 0.95 (IQR 0.77 - 1.17)] and SRU [0.97 (IQR 0.82 - 1.23)] values around the expected reference (1.00) (Figures 2B and 2D). In contrast, the SAPS 3-SE tended to place most ICUs below the reference, with a median SMR of 0.83 (IQR 0.67 - 1.02) and SRU of 0.90 (IQR 0.75 - 1.18) (Figures 2A and 2C). Hosmer-Lemeshow statistics were lower for the SAPS 3 Custom than for SAPS 3-SE. Sensitivity analyses by admission type (medical *versus* surgical) confirmed these findings for both discrimination and calibration, as well as for ICU performance and efficiency metrics (Table 2; Figure 2S [Supplementary Material]).

**Table 2:** Performance of SAPS-SE and SAPS-Custom

Score		Observed mortality %	Predicted mortality %	AUROC (95%CI)	H-L GOF (Chi-squared) *		SMR (95%CI)	SRU (95%CI)	Brier
					C-Statistics	H-Statistics			
All patients (n = 262,198)									
	SAPS 3-SE	16.4	19.0	0.841 (0.839 - 0.841)	1,739	1,776	0.86 (0.85 - 0.87)	0.90 (0.90 - 0.91)	0.102
	SAPS 3-Custom		16.6		339	327	0.98 (0.98 - 0.99)	0.98 (0.97 - 0.98)	0.100
Medical patients (n = 184,188)									
	SAPS 3-SE	19.5	23.0	0.825 (0.823 - 0.826)	1,815	1,837	0.85 (0.84 - 0.86)	0.92 (0.92 - 0.93)	0.118
	SAPS 3-Custom		20.1		337	246	0.97 (0.96 - 0.98)	1.00 (0.99 - 1.01)	0.117
Surgical patients (n = 78,010)									
	SAPS 3-SE	8.9	9.7	0.849 (0.844 - 0.849)	205	192	0.92 (0.90 - 0.94)	0.85 (0.84 - 0.86)	0.062
	SAPS 3-Custom		8.5		149	67	1.05 (1.02 - 1.07)	0.90 (0.90 - 0.91)	0.062

AUROC - area under the Receiver Operating Characteristic curve; 95%CI - 95%confidence interval; H-L GOF - Hosmer-Lemeshow goodness of fit test; SMR - standardized mortality rate; SRU - standardized resource use rate; SAPS - Simplified Acute Physiology Score.

* All p values < 0.001.

Patients were stratified into equal 10%-risk deciles (panels A and B) or number of patients per decile (panels C and D). The columns represent the number of patients per decile. The thin dotted line represents a perfect fit. The observed mortality in each decile is represented by blue diamonds with 95% confidence intervals.

The dotted, thin, and dashed lines represent the 95% and 99% confidence intervals, respectively. Using SAPS 3-SE, the median standardized mortality rate was 0.83 (IQR 0.67 - 1.02), and the median standardized resource use was 0.90 (IQR 0.75 - 1.18). Using SAPS 3-Custom, the median standardized mortality rate was 0.95 (IQR 0.77 - 1.17), and the median standardized resource use was 0.97 (IQR 0.82 - 1.23). SMR - standardized mortality rate; SRU - standardized resource use.

## DISCUSSION

In this study, we demonstrated that the recently proposed SAPS 3 customized equation provided accurate estimates of both hospital mortality and resource use in a large contemporary dataset from several ICUs in Brazil and Uruguay. On the other hand, SAPS 3-SE underestimated these outcomes. These findings have important implications for assessing and benchmarking ICU performance and efficiency in resource use in the present day.

The deterioration of SOIS performance over time has been described in several studies.^(22-25)^ Changes in case mix, admission and discharge policies, and patient management are expected to occur over time; therefore, the performance of any SOIS should be reassessed regularly. In general, a model's discrimination tends to remain relatively stable, whereas calibration can deteriorate, requiring recalibration to maintain accuracy.^(23,25)^ Periodical reassessments of the model's performance and eventual recalibration, when necessary, are regularly performed by well-established large ICU registries worldwide.^(9,23,26-28)^ Zimmerman et al. elegantly described the sequential customization of coefficients for the third version of the Acute Physiology and Chronic Health Evaluation (APACHE III) score, continuing until performance no longer met expectations, which ultimately led to the development of APACHE IV.^(23)^ However, first-level recalibration procedures are not always enough to improve SOIS accuracy appropriately.^(29,30)^ In such cases, recalibration using all original variables (second-level recalibration) may be attempted to further enhance calibration.^(31)^

In Brazil, the last extensive validation of SAPS 3-SE was conducted almost a decade ago.^(6)^ However, data from the *UTIs Brasileiras* registry had already demonstrated a trend toward progressively lower SMRs and SRUs in the years preceding the COVID-19 pandemic.^(5)^ While this trend could partly reflect genuine improvements in ICU performance, it also indicated the need to re-evaluate the accuracy of the SAPS 3-SE. The present study corroborates the findings of the *UTIs Brasileiras* white paper, confirming that the SAPS 3-SE is no longer accurate for outcome prediction. In contrast, first-level customization procedures improved calibration uniformity and the accuracy of resource use estimates. On the other hand, managers and researchers should be aware that once SAPS 3 is recalibrated, comparisons of mortality predictions and derived performance measures with those from other studies or registries will no longer be feasible.

Our study has many strengths. We used robust and appropriate statistical analyses to validate our results, and, to our knowledge, it is one of the most extensive SOIS validation studies to date. However, it also has some limitations. First, despite the large and heterogeneous number of units, our results cannot be interpreted as representative of all ICU patients in Brazil and Uruguay. Nevertheless, the ICU and patient characteristics in our dataset are comparable to those reported in the *UTIs Brasileiras* registry.^(5)^ Second, although the data were routinely collected by trained professionals for quality-improvement purposes, they were not audited, and missing data were expected (Table 1S - Supplementary Material). In addition, reporting biases are common in databases like ours, particularly for laboratory variables that are requested at the discretion of each participating ICU. Patients with a higher likelihood of abnormal results are more likely to undergo testing and, consequently, to have those results recorded. For this reason, we replaced missing data with normal values not only because the SAPS 3 investigators recommend this approach, but also because the missingness mechanism in our dataset was not at random (MNAR) (data not shown). Under such conditions, multiple imputation methods such as Multiple Imputation by Chained Equations (MICE) are not recommended.^(32)^ Third, we were unable to account for the impact of end-of-life decisions, as this information was not available. A prospective study capable of collecting such data while enrolling a comparable number of patients and ICUs would require an extensive multicenter effort. Finally, in the development of SAPS 3-Custom, the derivation dataset consisted of patients admitted in 2023 and 2024. In the present study, however, we used a convenience sample of patients admitted in 2022 and 2023. Therefore, validation studies in more contemporary patient populations are warranted.

## CONCLUSION

The SAPS 3-Custom was more accurate than the SAPS 3-SE in predicting outcomes and resource use in a large, contemporary cohort of intensive care unit patients in Brazil and Uruguay. These results provide strong evidence for adopting SAPS 3-Custom for performance and efficiency benchmarking in these countries. Nonetheless, as with any severity-of-illness scores, periodic re-evaluations are essential to ensure that it remains accurate in the future.

## Data Availability

Limited data that support the fundings of this study are available from the corresponding author, upon reasonable request.

## APPENDIX 1 ORCHESTRA Study Investigators

**Steering Committee:** Marcio Soares, Fernando Augusto Bozza; Jorge Ibrain Figueira Salluh; Pedro Martins Pereira Kurtz, Gastón Burghi.

**Research Coordinators:** Aline Reis da Silva Antunes, Grazielle Viana Ramos

**Statisticians:** Leonardo dos Santos Lourenco Bastos, Lunna Perdigão Borges, Thaís Machado, Gabriel Miranda

### Centers and Investigators

**Brazil: Alagoas -** *Hospital Memorial Arthur Ramos*: Maria Valéria de Carvalho Wanderley; Aline dos Santos Carvalho, Amanda Ribeiro de Mendonça Picone, Morghana Aparecida Rodrigues Ferreira, Bruna Xavier Brito; **Amazonas -** *Hospital Delphina Rinaldi Abdel Aziz*: Liane de Oliveira Cavalcante, Irina Jerez Jerez, Yudermys Amezaga Santana, Helena Alvarenga Sardenberg, Edna Freitas Martins, Marcia Lidiane Vasconcelos Dias Amorim; **Bahia -** *Hospital Cardiopulmonar*: Edson Silva Marques Filho; Antônio Fernando Borba Fróes Júnior, Daniel Beckerath da Silva Leitão; *Hospital Geral Cleriston Andrade:* Lúcio Couto de Oliveira Junior, Patrick Harrison Santana Sampaio, Renata Nunes de Oliveira; Diego Venicio Santos Argolo, Vanessa Freitas Vital, Bruno Cunha de Oliveira, João Victor Brito do Vale, Ramaiana de Jesus Gonzaga Cavalcante, Lúcio Couto de Oliveira Junior, Joaquim Agatângelo de Souza, Alberto Manoel Sarkis de Oliveira, Larissa Fernandes Oliveira, Daniela Cunha de Oliveira, Ricardo Peixoto Oliveira, Paulo Henrique Panelli Ferreira, Jamyllo Sales Brito, Elissama de Jesus Sena Reis, Vinicius Silva Oliveira, Geiza Santana Vidal; *Hospital da Cidade:* André Luiz Nunes Gobatto; Sydney Agareno de Souza Filho, Luciana Sampaio de Mattos Palmeira, Licurgo Pamplona Neto, Livia Magalhães Costa Castro; *Hospital Unimed Baia de Todos os Santos*: Lúcio Couto de Oliveira Junior; Joaquim Paulo Castro de Santana, Diego Venício Argolo, Patrick Harrison Sampaio, Joaquim Agatângelo Sousa, Tarsila Correia Ribeiro; **Ceará -** *Hospital Monte Klinikum*: David Theophilo Araújo; Victor Souza Cravo, Douglas Holanda Campos Filho, Ricardo Eustáquio Magalhães, Manuella Meireles Victor Souza Cravo, Pereira Gadelha Santos, Francisca Jane Gomes de Oliveira; **Distrito Federal**: *Hospital Anchieta:* Marcelo Oliveira Maia; Adriano Drummond, Noara Barros, Carla Moggia, Ivna Asfor; *Hospital Santa Luzia:* Marcelo Oliveira Maia; Fábio Amorim, Carlos Darwin; *Hospital DF Star*: Antônio Aurélio Fagundes Jr; *Hospital Brasília Unidade Águas Claras*: Vinicius Machado Santos; Pedro Henrique Rosa da Silveira, Tiago Samuel Lima Pontes, Flavio Carvalho dos Santos; **Espírito Santo -** *Hospital Meridional:* Marcus Vinicius A. Leitão; Lucas Resende Aniceto, Lucas Dornelas F. Machado Silva, Shayra Pansini Souza, Frederico Machado de Siqueira; *Hospital Unimed Vitória*: Eliana Bernardete Caser; **Goiás -** *Hospital Municipal de Aparecida de Goiânia – HMAP*: Joan Rodrigues de Castro; Maurício Mascarenhas Boaventura, Cristian Andrade Garcia, Renata de Souza Cyrino, Gean Carlos Alves Moraes; *Hospital Israelita Albert Einstein – Goiânia*: Ângelo Antônio Gomes de Carvalho; *Centro Estadual de Reabilitação e Readaptação Dr. Henrique Santillo – CRER*: Eduardo Vilela, Ciro Bruno Silveira Costa, Priscila Martins Pereira, Ludmila Gomes dos Santos, Ronycley Rocha Rezende, Fabianne Silveira Cardoso; *HUGOL – Hospital Estadual de Urgências Governador Otávio Lage de Siqueira*: Antônio Elias Lopes, Flávio Augusto Castro, Gustavo Prudente, Alex Linhares, Gabriel Fogaça, Igor Ferreira Capelleti; **Maranhão** - *Hospital São Domingos:* José Raimundo Azevedo; Luis Eduardo França Tupinambá Junior; *UDI Hospital*: Alexandre Guilherme Ribeiro de Carvalho; Edilene Coelho de Souza Novaes, Lucas Akira Costa Hirai, Daniel Wagner de Castro Lima Santos, Louise Aline Romão Gondim, Tânia Karla Sousa Nogueira Rosa; *Hospital Maranhense:* Filipe Sousa Amado; *Hospital do Cancer do Maranhão Tarquinio Lopes Filho*: Gustavo Teixeira Alves; Sara Vieira Nascimento; *Hospital de Traumatologia e Ortopedia do Maranhão*: Luciana Sousa Silva; Rosinete Andrade Ferreira; **Minas Gerais** - *Hospital Metropolitano Doutor Célio de Castro*: Luidy Luciano Cardoso; Roberto Sydney, Paolo Tótola, Bruno Resende, Diogo Madeira; *Hospital Felício Rocho*: Rogério de Castro Pereira; Thais de Paula Guimarães, Sinval Lins Silva, Daniel Fontes, Débora Avelar Afonso da Silva, Ana Clara Ferreira Amâncio Pereira; *Hospital das Clínicas da UFMG*: Saulo Fernandes Saturnino; *Pró-Saúde Hospital Metropolitano Vale do Aço:* Luiz Henrique de Araújo Pereira Costa; Norberto de Sá Neto, Rodrigo Silveira Machado, José Roberto Batista; *Hospital Risoleta Tolentino Neves*: Marco Aurélio Reis, Pulchéria Leôncio Pereira Araujo, Camila Martins Ramos; **Pará -** *Hospital Adventista de Belém*: Edgar de Brito Sobrinho; **Paraíba -** *Hospital Nossa Senhora das Neves*: Paulo César Gottardo; Elbia Assis Wanderley, Katyucia Egito de Araújo Urquisa, Andreia Cristina Fumagalli Cainelli, Vinilton Leandro Ferreira; *Hospital Universitário Lauro Wanderley - UFPB/EBSERH*: Ciro Leite Mendes; Igor Mendonça do Nascimento; **Paraná -** *Hospital Santa Cruz*: Hipolito Carraro Jr. *Hospital São Marcelino Champagnat*: Viviane Bernardes de Oliveira Chaiben; Maria Lygia Minney, Andressa de Souza Bertoldi, Karen Fernandes de Moura, Gustavo Henrique dos Santos Silva, Bruno Alcantara Gabardo; *Hospital Municipal Padre Germano Lauck*: Roberto de Almeida; Guilherme Ribeiro, Mirian Liliana Insfrán Franco; *Hospital Universitário Cajuru*: Viviane Bernardes de Oliveira Chaiben; Giovanna Cerri Lessa, Victor Hugo Santana Lourenço de Lima, Leandro Bressianini Jurkonis, Marcos vinicius Streit, Gabriela Martins Teixeira; *Hospital Nossa Senhora das Graças:* Iara Buselato Chen; Vanessa Padilha Tomba, Marta Ângela Brandão; *Hospital Araucária de Londrina*: Cintia Magalhães Carvalho Grion; *Hospital Universitário Regional do Norte do Paraná*: Cintia Magalhães Carvalho Grion; **Pernambuco -** *Real Hospital Português*: Mário Diego Teles Correia; *Hospital Santa Joana Recife*; Gustavo Trindade Henriques Filho; Arthur Henrique Ribeiro do Valle de Faria, Marcos Antonio Cavalcanti Gallindo, Carlos Eduardo Ferraz Freitas, Ana Flávia de Melo Campos, Danielle Ferraz de Oliveira Aguiar; **Rio de Janeiro -** *Hospital Copa D’Or:* Aloysio Saulo Beiler; *Hospital Quinta D’Or*: Alexandre Coscia, Laura Herranz Prinz, Juliana Gurgel da Silveira, Cristiane Belo, Soraya Pulier; Joao Vitor Bessa, Juliana Gravina, Thiago Prata, Joyce Roma Lucas de Silva, Alessandra Longo, Lorena fonseca, Alexandra Gonçalves, Marcelo Cruzick; *Hospital Estadual Alberto Torres*: Ulisses de Oliveira Melo; *Hospital Nossa Senhora do Carmo*: Paula Figueiredo Natel; Raphaela Mannarino, Nelson Poubel, Amir Gonçalves Neto, Marcelo Motta; *Complexo Américas*: Victor Cravo; Emmanuel Salgueiro; *Hospital Norte D’Or*: Douglas Quintanilha Braga; Sergio Teixeira Sant’Anna Júnior, Gustavo Caniné da Costa, Renata Ribeiro Leite do Amaral, Camila Lima Ferreira da Costa, Thalita Montenegro Prieto Lloret; *Hospital Barra D´Or*: Walter Homena Jr, Marcelo de Sousa Santino, Gloria Adriana Rocha Martins; José Alexandre Espósito Panaro, Francisco Gonçalves Gabriel, Paula Gorgulho, Marcelo Felix, Luciana Freitas de Oliveira; *Hospital Pasteur*: Pedro Henrique Barbosa D’ Almeida; Carlos Eduardo Brandão, Raquel Pereira De Farias Evangelista, Danessa Moreira Rodrigues; *Hospital Rios D’Or*: Alessandra Alves; *Hospital Caxias D’Or*: Eric Perecmanis; *Hospital Niterói D’Or*: Ricardo Turon Bruno Guimarães; *Hospital Oeste D’Or*: Guilherme Brenande Alves Faria, Márcia Adélia de Magalhães Menezes; Liliane Rodrigues de Mendonça, Reinaldo Campos Rodrigues, Caros Henrique Ferreira Ramos, Rosa Imaculada Stancato, Joyce Andrade; *Hospital São Lucas Copacabana*: Aline Affonso, Laura Herranz Prinz; Bruno Gonçalves; *Hospital Municipal Evandro Freire*: Fabio Basilio Fernandes dos Santos; *Hospital Adventista Silvestre*: Fernando Santiago Montenegro; *Hospital Badim*: Edmundo de Oliveira Tommasi; Alexandra Goncalves da Silva, Alexandre Vaz Scotti, Jefferson dos Santos Daros, Fábio Guilherme Santoro; *Hospital Samer:* Henrique Miller Balieiro; Marcelo Namen; *Hospital Copa Star:* Pedro Kurtz, Rodolfo Espinoza; Rafaella Pottes; *Hospital de Clínicas de Jacarepaguá*: Fernando Alves Rocha; Simone Cristina Santos De Lira, Ana Paula Figueiredo De Carvalho, Carlos Eduardo Brandão; *Hospital Gloria D’Or*: Cecília Magno; Nathane Santanna; *Hospital Municipal da Japuiba*: Viviane Bogado Leite Torres; Michelle Cristina Ferreira Soares, Beatriz Victoria Correia Ferreira, Josiane Cristina da Silva, Julia Monteiro Novaes, Tatiany Lopes Lessa; *Hospital de Clínicas Mario Lioni*: Fernando Santana Pinto; Carlos Eduardo Brandão, Juliana Sales De Ornelas; *Hospital e Maternidade Santa Lúcia:* Eduardo Costa Pinto; Carlos Eduardo Brandão, Elen Silva Ferreira, Marcelo Alonso De Barros Correia; *Hospital da Fundação Eletronuclear de Assistência Médica – FEAM*: Viviane Bogado Leite Torres, Mariana Rodrigues Farias Andrade; *Instituto Nacional de Câncer - Hospital do Câncer II:* Rodolfo Espinoza, Rafael Mandarino; **Rio Grande do Sul -** *Hospital Mãe de Deus*: Lucas Vieira de Souza; *Hospital de Clínicas de Porto Alegre:* Fabiano Márcio Nagel; Márcio Manozzo Boniatti; *Hospital Dom João Becker*: Michael Milman; **Santa Catarina -** *Hospital Municipal Ruth Cardoso*: Pedro Salomão Dias, Cesar Augusto Meirelles de Almeida, Eduardo Bellotto, Pablo Wanglon Richter; **São Paulo –** *Hospital A.C. Camargo*: Antônio Paulo Nassar Jr; Silvana Soares dos Santos; *Hospital e Maternidade Brasil:* Fabio de Carvalho Mauricio; Tatiana Gozzi Pancev Toledo, Fernando Ramos Pellegrini, Manoela Prado Pasqualucci Esposito; *Hospital Municipal Moysés Deutsch*: Niklas Soderberg Campos; Bruna Achar Soderberg Campos, Paula Geraldes David João, Luiz Adriano Esteves, Petrus Söderberg Campos, Luciana Silveira de Oliveira; *Hospital Nove de Julho*: Marcos Soares Tavares; Celso Madeira Padovesi, Antônio Paulo Martins Ramos Filho; *IAMSPE - Hospital do Servidor Público Estadual*: Ederlon Alves de Carvalho Rezende; Ellen Pierre Oliveira, Caio Gouvêa Jaoude, Vânia Quinato Malacize, Mateus Demarchi Gonsalves; *Hospital de Base*: Suzana Ajeje Margareth Lobo; Luana Fernandes Machado, Juliana Devós Syrio Martinez, Marcio Mussolino de Queiroz, Neymar Elias de Oliveira, Silvia Prado Minhoto Teixeira Ramin, Vanessa Aparecida Maziero Santana; *Hospital São Luiz Morumbi*: José Célio Vieira Brandão; Adriana Peixoto Gelmetti de Barros; *Hospital ViValle*: Flavio Rodrigues de Sousa; Felipe de Jesus Gonçalves; *Hospital São Luiz Itaim*: Mariza Silva Ramos Loesch, Thiago Gomes Romano; *Hospital Villa-Lobos*: Carlos Antônio Carvalho Ribeiro; Flávio Geraldo Rezende de Freitas; *Hospital Israelita Albert Einstein – Morumbi*: Thiago Domingos Corrêa; *Hospital do Coração HCOr*: Edson Renato Romano; Rosianne Vasconcelos; *Hospital Assunção*: Silvia Regina Ramos; *Hospital Metropolitano*: Eduardo Augusto Pessoa Gomes; Carlos Eduardo Brandão, Liliane Alves Feitoza Turci, Aline Nunes Lobo Bueno; *Hospital da Luz*: Daniel Almeida Schettini; Carlos Eduardo Brandão, Vaneska Mazzini, Lilian Louise Coelho Pereira; *Hospital Sírio Libanês - Bela vista SP:* Laerte Pastore Jr.; Fernando José da Silva Ramos, Naira Lima Matos, Clara Esther Maciel dos Santos, Bruno Martins Tomazini, Nilda Rosa de Oliveira Prado; *Hospital Panamericano*: Eduardo Costa Pinto; Carlos Eduardo Brandão, Vanessa Ribeiro Pardauil, Cristiane Cunha Da Silva Rosa; *Hospital Vitória Anália Franco*: Guilherme Rossini; Carlos Eduardo Brandão, Mariana Celeghini Santiago Gosik, Priscilla Belchior Gonzalez; *Serviço Social da Ind. do Papel Papelão e Cortiça Do Est. São Paulo - Hospital e Maternidade SEPACO:* Nathaly Fonseca Nunes; Daniela Boschetti, Flávio Geraldo Rezende de Freitas; *Hospital Alvorada Moema*: Amanda Mota de O. Veiga; Carlos Eduardo Brandão; *Hospital Carlos Chagas*: Fernando Jose Bricks; Carlos Eduardo Brandão, Caroline Scodelario Cortes, Francisco Afranio Miranda; *Hospital São Paulo*: Flavia Ribeiro Machado; *Hospital Santa Helena*: Alessandra Borges Mendes Gonzaga; Carlos Eduardo Brandão, Tatiane Oliveira Luiz, Liliane Lemos; *Hospital Japonês Santa Cruz:* Thiago Miranda Lopes de Almeida; Sérgio Sônego Fernandes, Carlos Antônio Carvalho Ribeiro, Flávio Geraldo Rezende de Freitas; *Hospital Samaritano Higienópolis*: Marcos Cairo Vilela; Barbara Cristina de Abreu Pereira, Fernando Antonio Alvares da Costa, Leonardo Fernando Ferrari Nogueira, Natalia Lopes Ferreira, Luciana Rosa Fidelis; *BP – A Beneficência Portuguesa de São Paulo – Unidade Mirante*: Viviane Cordeiro Veiga; *Hospital Ana Costa*: Fernanda Rodrigues Martins Masteguim; Carlos Eduardo Brandão, Thiago Santos da Silva, Fatima Cristina Andrade Rodrigues; *Hospital Paulistano*: Alder Costa Garcia da Silveira; Carlos Eduardo Brandão, Debora Prudencio E Silva, Helenice De Paula Vieira; *Santa Casa de Piracicaba*: Rafael Angelo Tineli; Luciana Marcolino Tineli; *Hospital Beneficência Portuguesa de Ribeirão Preto*: Marcus Antônio Ferez; Franceliana Prado Barbosa Sgobi, Fernanda Valéria Ramos Paiolo; *Hospital e Maternidade Ribeirão Pires*: Fabio de Carvalho Mauricio, Tatiana Gozzi Pancev Toledo, Fernando Ramos Pellegrini, Manoela Prado Pasqualucci Esposito; *Hospital dos Fornecedores de Cana de Piracicaba*: Renata Lopes Basso; Rafael Angelo Tineli; *Hospital Pitangueiras*: Márcio Shimabuku e Silva; Carlos Eduardo Brandão, Felipe Neves Marcelino, Alex Oliveira; *Hospital Vila Nova Star:* Thiago Gomes Romano; *Hospital São Bernardo:* Ana Paula Mascarelli Amaral; Carlos Eduardo Brandão, Maria De Fatima Silva De Almeida, Janaina De Cassia Lopes Dos Santos; *Hospital Novo Atibaia*: Rubens Sergio da Silva Franco; Amauri Francisco De Marchi Bemfica, Walter Carlos Girardelli Baptista, Manoela Moreira de Sousa, Aline Ribeiro Moreira, Juliana Regina Berto Wada; *Hospital de Clinicas de Caieiras*: Volnei Martins Castanho; Carlos Eduardo Brandão, Priscila Gonzaga dos Santos, Elaine Santana da Silva, Marciely Alves Ramalho de Oliveira, Viviane Gimenez Rossini, Imara Jacinto de Azevedo Rios; *Hospital Ipiranga*: Rafael Di Domenico Mattos; Carlos Eduardo Brandão, Fabricio Campos Morais Moreira, Julienne Garcia Rissatti, Luiz Henrique Costa Garcia; *Hospital Santo Amaro:* Sanmya Danielle Rodrigues dos Santos; Carlos Eduardo Brandão, Dominique Almeida Cruz, Bárbara Fialdini Von Ah; *Hospital e Maternidade Ipiranga Arujá*: Rafael Di Domenico Mattos, Carlos Eduardo Brandão, Gisele Aparecida Cardoso; *Hospital das Clínicas da Faculdade de Medicina da Universidade de São Paulo*: Leandro Utino Taniguchi; Bruno Adler Maccagnan Pinheiro Besen; Roberta Muriel Longo Roepke; Pedro Fortes Osório Bustamante; Ana Clara Marcondes Dobre; *Unimed Capivari*: Rafael Angelo Tineli; Daniela Mazzini Quagliato Azanha; *Hospital IFOR*: Sílvia Regina Ramos.

**Uruguay - Montevideo -** *Hospital Maciel*: Gastón Burghi; *Sanatório Americano*: Gastón Burghi; Pedro Alzugaray; *COMECA:* Gastón Burghi; Carlos Pan; *Hospital Policial:* Pedro Saldun; Gonzalo Lacuesta, Sergio Rovira, Lourdes Ferro, Silvana Lopez; *Hospital Evangélico*: Gastón Burghi; *COMERO IAMPP*: Andrés Cebey; Carlos Cardoso; *CRAMI*: Gastón Burghi; Pedro Alzugaray; *CAMOC*; Gastón Burghi; Pedro Alzugaray

## References

[1] Salluh JI, Soares M (2014). ICU severity of illness scores: APACHE, SAPS and MPM. Curr Opin Crit Care.

[2] Keegan MT, Gajic O, Afessa B (2011). Severity of illness scoring systems in the intensive care unit. Crit Care Med.

[3] Keegan MT, Soares M (2016). What every intensivist should know about prognostic scoring systems and risk-adjusted mortality. Rev Bras Ter Intensiva.

[4] Moreno RP, Metnitz PG, Almeida E, Jordan B, Bauer P, Campos RA, SAPS 3 Investigators (2005). SAPS 3—From evaluation of the patient to evaluation of the intensive care unit. Part 2: development of a prognostic model for hospital mortality at ICU admission. Intensive Care Med.

[5] Associação de Medicina Intensiva Brasileira, Epimed Solutions UTIs Brasileiras. Adult ICU Profile.

[6] Moralez GM, Rabello LS, Lisboa TC, Lima MD, Hatum RM, De Marco FV, ORCHESTRA Study Investigators (2017). External validation of SAPS 3 and MPM_0_-III scores in 48,816 patients from 72 Brazilian ICUs. Ann Intensive Care.

[7] Maccariello E, Valente C, Nogueira L, Bonomo H, Ismael M, Machado JE (2010). SAPS 3 scores at the start of renal replacement therapy predict mortality in critically ill patients with acute kidney injury. Kidney Int.

[8] Soares M, Silva UV, Teles JM, Silva E, Caruso P, Lobo SM (2010). Validation of four prognostic scores in patients with cancer admitted to Brazilian intensive care units: results from a prospective multicenter study. Intensive Care Med.

[9] Ferrando-Vivas P, Jones A, Rowan KM, Harrison DA (2017). Development and validation of the new ICNARC model for prediction of acute hospital mortality in adult critical care. J Crit Care.

[10] Soares M, Dongelmans DA (2017). Why should we not use APACHE II for performance measurement and benchmarking?. Rev Bras Ter Intensiva.

[11] Rothen HU, Stricker K, Einfalt J, Bauer P, Metnitz PG, Moreno RP (2007). Variability in outcome and resource use in intensive care units. Intensive Care Med.

[12] Zampieri FG, Soares M, Salluh JI (2020). How to evaluate intensive care unit performance during the COVID-19 pandemic. Rev Bras Ter Intensiva.

[13] Associação de Medicina Intensiva Brasileira, Epimed Solutions UTIs Brasileiras.

[14] Soares M, Salluh JI, Zampieri FG, Bozza FA, Kurtz PM (2024). A decade of the ORCHESTRA study: organizational characteristics, patient outcomes, performance and efficiency in critical care. Crit Care Sci.

[15] Ferreira JC, Pereira AJ, Cavalcanti AB, Nassar AP, Serpa A, Besen BA (2025). Brazilian Research in Intensive Care Network (BRICNet): shaping the landscape of critical care research in Brazil and beyond. Crit Care Sci.

[16] Soares M, Borges LP, Bastos LD, Zampieri FG, Miranda GA, Kurtz P (2024). Update on the Epimed Monitor Adult ICU Database: 15 years of its use in national registries, quality improvement initiatives and clinical research. Crit Care Sci.

[17] Vincent JL, Moreno R, Takala J, Willatts S, De Mendonça A, Bruining H (1996). The SOFA (Sepsis-related Organ Failure Assessment) score to describe organ dysfunction/failure. On behalf of the Working Group on Sepsis-Related Problems of the European Society of Intensive Care Medicine. Intensive Care Med.

[18] Charlson ME, Pompei P, Ales KL, MacKenzie CR (1987). A new method of classifying prognostic comorbidity in longitudinal studies: development and validation. J Chronic Dis.

[19] Lemeshow S, Hosmer DW (1982). A review of goodness of fit statistics for use in the development of logistic regression models. Am J Epidemiol.

[20] The R Foundation R: The R Project for Statistical Computing.

[21] EQUATOR Network Enhancing the QUAlity and Transparency Of Health Research [Internet].

[22] Soares M, Salluh JI (2006). Validation of the SAPS 3 admission prognostic model in patients with cancer in need of intensive care. Intensive Care Med.

[23] Zimmerman JE, Kramer AA, McNair DS, Malila FM (2006). Acute Physiology and Chronic Health Evaluation (APACHE) IV: hospital mortality assessment for today's critically ill patients. Crit Care Med.

[24] Genu DH, Lima-Setta F, Colleti J, de Souza DC, Gama SD, Massaud-Ribeiro L, Brazilian Research Network in Pediatric Intensive Care (BRnet-PIC) (2022). Multicenter validation of PIM3 and PIM2 in Brazilian pediatric intensive care units. Front Pediatr.

[25] Davis SE, Lasko TA, Chen G, Matheny ME (2018). Calibration drift among regression and machine learning models for hospital mortality. AMIA Annu Symp Proc.

[26] Paul E, Bailey M, Kasza J, Pilcher DV (2017). Assessing contemporary intensive care unit outcome: development and validation of the Australian and New Zealand Risk of Death admission model. Anaesth Intensive Care.

[27] Bruserud Ø, Haaland ØA, Kvåle R, Buanes EA (2023). A first-level customization study of SAPS II with Norwegian Intensive Care and Pandemic Registry (NIPaR) data. Acta Anaesthesiol Scand.

[28] Metnitz B, Schaden E, Moreno R, Le Gall JR, Bauer P, Metnitz PG, ASDI Study Group (2009). Austrian validation and customization of the SAPS 3 Admission Score. Intensive Care Med.

[29] Cox EG, Wiersema R, Eck RJ, Kaufmann T, Granholm A, Vaara ST (2023). External validation of mortality prediction models for critical illness reveals preserved discrimination but poor calibration. Crit Care Med.

[30] Kurtz P, Bastos LS, Salluh JI, Bozza FA, Soares M (2021). SAPS-3 performance for hospital mortality prediction in 30,571 patients with COVID-19 admitted to ICUs in Brazil. Intensive Care Med.

[31] Moreno R, Apolone G (1997). Impact of different customization strategies in the performance of a general severity score. Crit Care Med.

[32] Hughes RA, Heron J, Sterne JA, Tilling K (2019). Accounting for missing data in statistical analyses: multiple imputation is not always the answer. Int J Epidemiol.

