# Supplementary material for: Contemporary validation of a SAPS 3 customized version in patients admitted to Brazilian and Uruguayan intensive care units: a multicenter cohort study
Source: Crit Care Sci. 2026 Mar 16;38:e20260334. doi: 10.62675/2965-2774.20260334 (PMC13124105; doi:10.62675/2965-2774.20260334)
Supplement: Supplementary Material [file 2965-2774-ccsci-38-e20260334-suppl.pdf]

Critical Care Science
https://doi.org/10.62675/2965-2774.20260334

SUPPLEMENTARY MATERIAL

Contemporary validation of a SAPS 3 customized version in patients admitted to Brazilian and Uruguayan intensive care units: a multicenter cohort study

Marcio Soares[1], Lunna Perdigão Borges[2], Gastón Burghi[3], Pedro Kurtz[1], José Raimundo Araújo de Azevedo[4], Carlos Eduardo Brandão[5], Aloysio Saulo Breves Beiler Júnior[6], Niklas Soderberg Campos[7], Liane Oliveira Cavalcante[8], Mario Diego Teles Correia[9], Victor de Souza Cravo[10], Pedro Henrique Barbosa D'Almeida[11], Flávio Geraldo Rezende de Freitas[12], Thais de Almeida Machado[2], Marcelo de Oliveira Maia[13], Edson Silva Marques Filho[14], Gloria Adriana Rocha Martins[1], Ulisses de Oliveira Melo[15], Laura Herranz Prinz[16], Silvia Regina Ramos[17], Thiago Gomes Romano[18], Marcos Soares Tavares[19], Suzana Margareth Lobo[20], Jorge Ibrain Figueira Salluh[1], Ederlon Rezende[21], on behalf of the ORCHESTRA Study Investigators

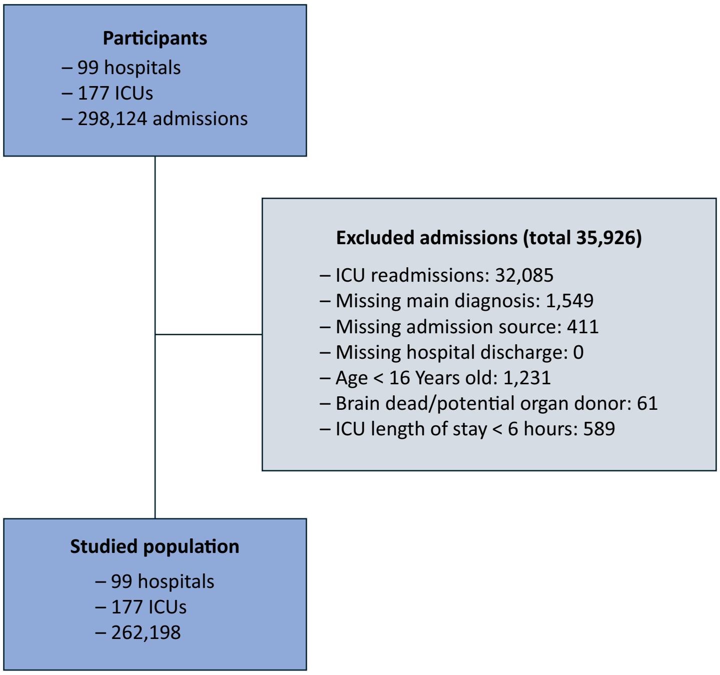

Figure 1S - Study flowchart.
ICU - intensive care unit.

This is an open access article under the CC BY license (https://creativecommons.org/licenses/by/4.0/).

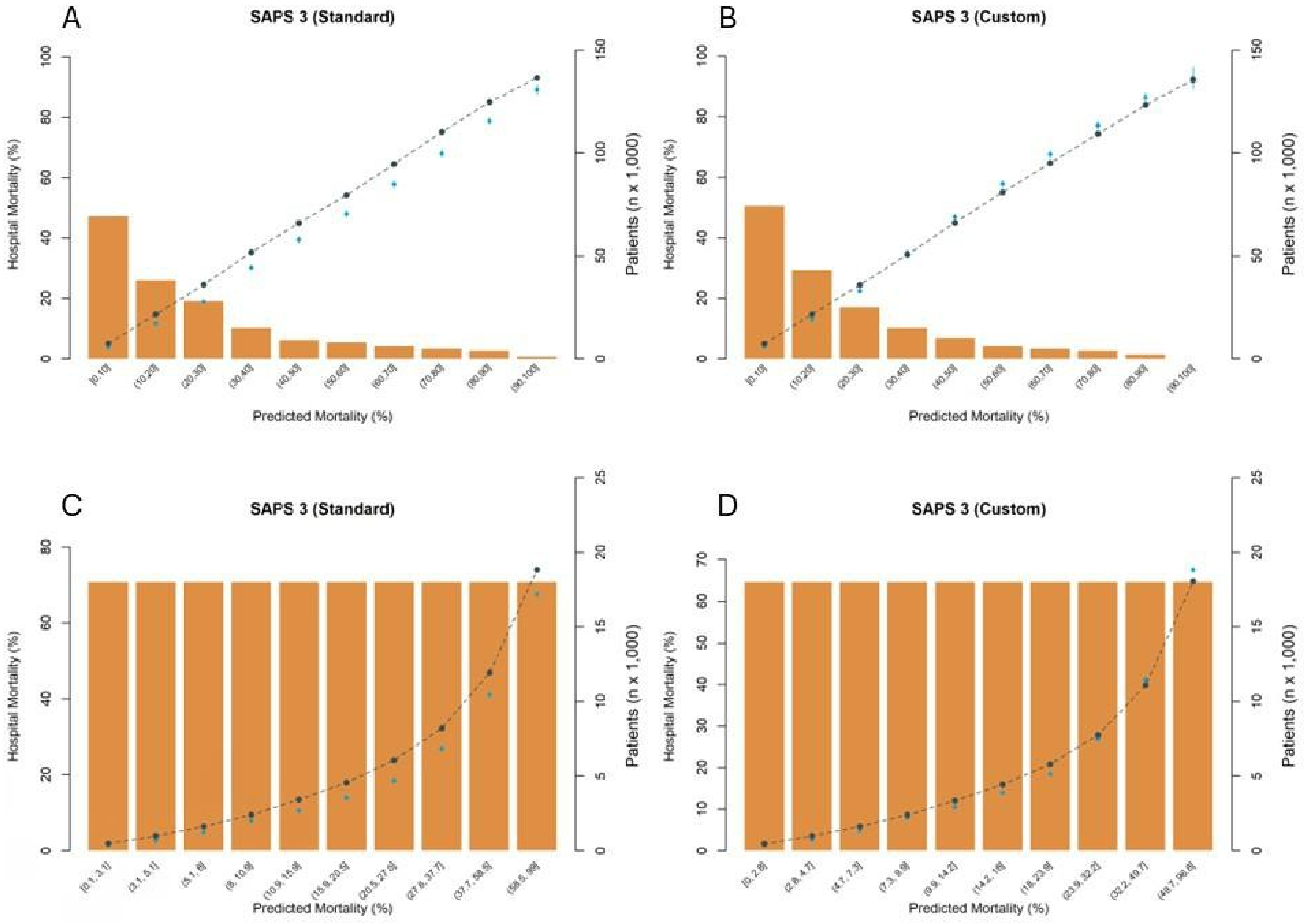

Figure 2S - Calibration curves for the standard (SAPS 3-SE) and customized (SAPS 3-Custom) equations in medical admissions (n =184,188).
Patients were stratified into equal 10%-risk deciles (panels A and B) or number of patients per decile (panels C and D). The columns represent the number of patients per decile. The thin dotted line represents a perfect fit. The observed mortality in each decile is represented by blue diamonds with 95% confidence intervals.

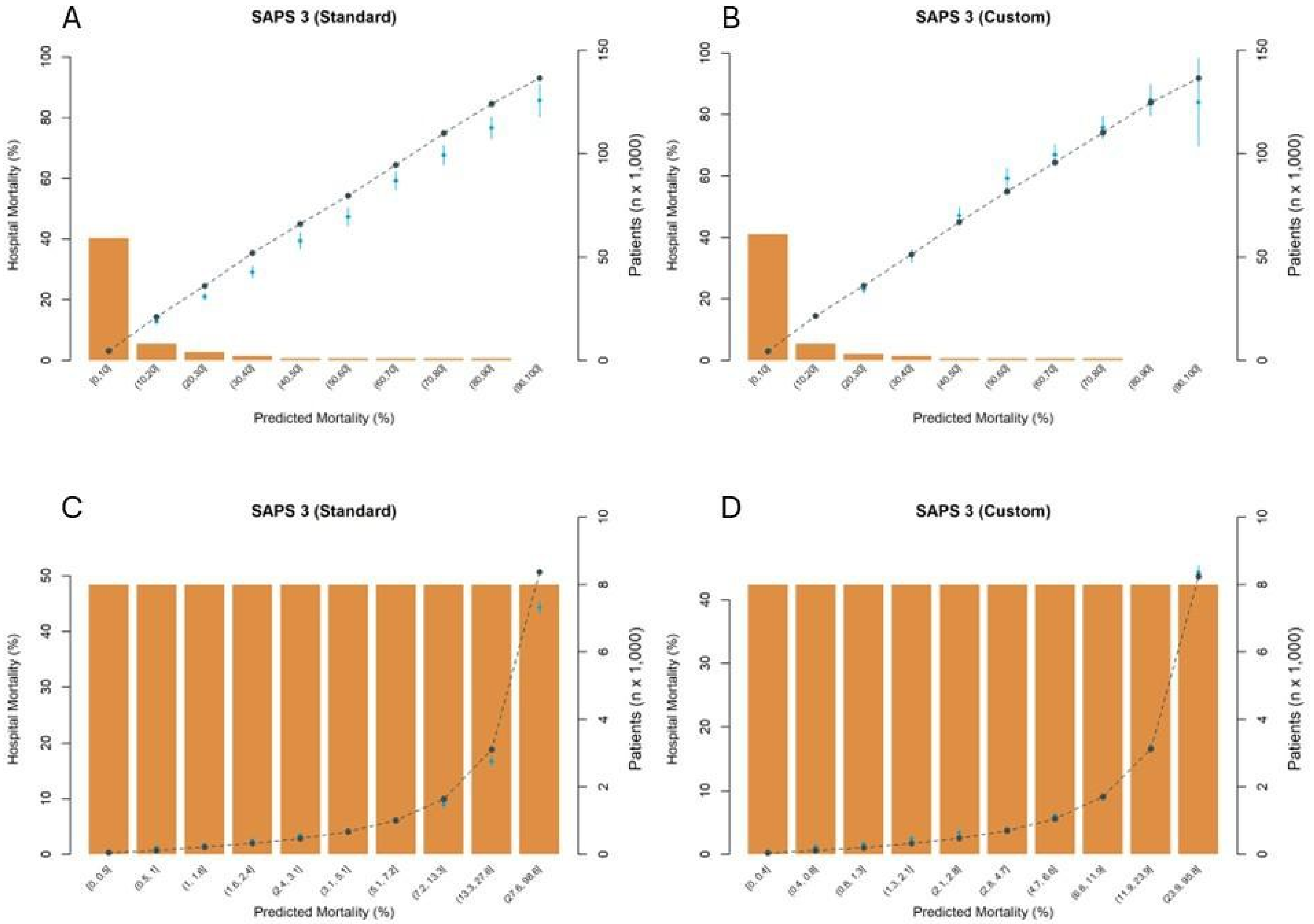

Figure 3S - Calibration curves for the standard (SAPS 3-SE) and customized (SAPS 3-Custom) equations in surgical admissions (n = 78,010).
Patients were stratified into equal 10%-risk deciles (panels A and B) or number of patients per decile (panels C and D). The columns represent the number of patients per decile. The thin dotted line represents a perfect fit. The observed mortality in each decile is represented by blue diamonds with 95% confidence intervals.

Table 1S - SAPS 3 missing variables (n = 262,198)

Missing variables	n (%)
Age	0
Comorbidities	1,802 (0.60)
Admission source	1,875 (0.63)
Length of hospital stay before ICU admission	241 (0.08)
Vasoactive drugs before ICU admission	2,611 (0.88)
SAPS 3 admission diagnoses	1,906 (0.64)
Admission type	1,779 (0.60)
Surgical status	1,902 (0.64)
Acute infection	2,732 (0.92)
Systolic blood pressure	8,967 (3.01)
Heart rate	8,920 (2.99)
Body temperature	19,147 (6.42)
Glasgow coma scale	26,586 (8.92)
Leukocyte	35,463 (11.90)
Creatinine	36,188 (12.14)
Platelets	35,250 (11.82)
Total bilirubin	159,975 (53.66)
pH	165,457 (55.50)
PaO2	168,331 (56.46)
PaO_2/FiO_2	112409 (67.31)
Ventilatory support at admission	2,611 (0.88)
PaO_2 in ventilated patients	10,736 (23.55)
PaO_2/FiO_2 in ventilated patients	14,064 (30.85)

ICU - intensive care unit; SAPS - Simplified Acute Physiology Acute Score; PaO_2 - arterial oxygen partial pressure; FiO_2 - fractional inspired oxygen. Mechanical ventilation at admission (± 1 hour; n: 38,559).

Table 2S - Length of stay per surviving patient according to the SAPS-SE and SAPS 3-Custom for the purposes of standardized resource use rate estimation (n = 262,198)

SAPS 3 classes	SAPS 3 (points)	Patients (n)	Survivors (n)	Original LOS per survivor (days)	Customized LOS per survivor (days)
1	0 - 24	14,087	13,976	2.3	2.39
2	25 - 34	49,531	48,555	3.2	2.99
3	35 - 44	64,940	61,491	4.3	4.14
4	45 - 54	63,759	55,225	7.2	6.00
5	55 - 64	36,937	26,614	11.0	9.45
6	65 - 74	17,298	8,973	16.6	16.12
7	75 - 84	8,889	3,105	22.2	26.10
8	85 - 94	4,407	1,014	29.4	37.34
9	≥ 95	2,350	311	39.0	57.14

SAPS 3 - Simplified Acute Physiology Score 3; LOS - length of stay. Average number of expected length of stay per survivor for the purpose of standardized resource use rate estimation according to the SAPS-SE and SAPS 3-Custom. We stratified our patient population into the nine SAPS 3 classes (< 24; 25 - 34; 35 - 44; 45 - 54; 55 - 64; 65 - 74; 75 - 84; 85 - 94; ≥ 95 points) as originally proposed by Rothen et al.[11]

Table 3S - Main intensive care unit characteristics (n = 177)

Characteristic	
Hospital type	
Public	63 (35.6)
Private	114 (64.4)
Unit type	
Medical-surgical	136 (76.8)
Surgical	12 (6.9)
Neurological	10 (5.7)
Oncological	9 (5.1)
Medical	7 (4.0)
Other	3 (1.7)
No. of ICU beds	
< 10	20 (11.3)
10 - 19	101 (57.1)
20 - 29	36 (20.3)
≥ 30	20 (11.3)

ICU - intensive care unit. Results expressed as n (%).

SUMMARY OF CUSTOMIZED SAPS 3 MODEL DEVELOPMENT REPORTED IN THE "WHITE PAPER" PERFORMED BY THE STEERING COMMITTEE OF THE *UTIS BRASILEIRAS* REGISTRY.

Available at: https://www.utisbrasileiras.com/en/

STUDY DESIGN AND SETTING

Retrospective analysis of prospectively collected data from patients admitted to adult ICUs participating in the Brazilian ICU Registry between January 1st, 2023 and September 30th, 2024.

THE BRAZILIAN ICUS REGISTRY[1]

The registry is an initiative led by AMIB in partnership with Epimed Solutions® (Rio de Janeiro, Brazil) to characterize the epidemiological profile of Brazilian ICUs and share useful information to guide health policies and strategies to improve the care of critically ill patients in the country. Approximately 50% of all adult ICUs from Brazil participate in the Registry.

PARTICIPANTS, DATA COLLECTION AND DEFINITIONS

Intensive care units: 1,239 adult ICUs with ≥ 5 beds in in 562 hospitals. Intensive care units with > 60% of patients missing admission diagnosis or > 20% of patients transferred to another hospital/institution, nursing facilities or hospice at hospital discharge were excluded.

Patients: 1,306,811 adult patients admitted during the study period. All adult patients (≥ 16 years old), excluding potential organ donors and brain dead at ICU admission, ICU readmissions during the same hospitalization, ICU length of stay (LOS) < 1 hour and missing hospital discharge data.

Data collection: data collection was a varying combination of integration with the hospital's electronic medical and/or administrative records (HER) and manual data entry depending on the hospitals' information technology infrastructure. In most ICUs, administrative (demographics, ICU and hospital admission/discharge information) are integrated, and a dedicated case manager

(usually nurses) is responsible for entering the remaining clinical and laboratory data for every consecutive patient into the database.

Variables: data included demographics, admission source, hospital LOS before ICU admission, primary ICU admission diagnosis, Sequential Organ Failure Assessment (SOFA) score at admission,[2] comorbidities based on the Charlson Comorbidity Index,[3] frailty assessed by the Modified Frailty Index,[4] use of organ support during the ICU stay, ICU and hospital LOS, vital status at hospital discharge (dead or alive) and destination after hospital discharge.

Outcomes: the primary outcome was all-cause in-hospital mortality at the patient level. The ICU LOS was the secondary outcome.

STATISTICAL ANALYSIS

Missing data handling: as recommended by the SAPS 3 calculation, normal values were input for laboratory and physiological variables.

Descriptive statistics of ICU and patient characteristics: continuous variables were expressed as the means ± standard deviation or medians (25% - 75% interquartile range, IQR), as appropriate. Categorical variables were expressed as absolute numbers (frequency percentages).

Calculation of SAPS 3 standard parameters: the logit [-32.6659 + ln(SAPS 3 score + 20.5958) × 7.3068] were calculated to estimate the probability of death: elogit/(1+elogit).[5] The number of ICU days to produce a survivor estimated by Rothen et al. was used as reference to estimate resource use.[6]

Intensive care unit performance indicators: standardized mortality (SMR) and resource use (SRU) ratios with 95% CIs were estimated to evaluate clinical performance and resource use efficiency, respectively. The SMR is the ratio between the observed and predicted hospital mortality. The SRU estimates the average observed-to-expected ratio of resources (based on the ICU LOS) used per surviving patient in a specific ICU adjusted for the SAPS 3 (please, refer to Table 2S of this ESM).

Assessment of model's performance: discrimination (i.e., the ability of each model to discriminate between patients who lived and patients who died) by estimating the area under the receiver operating characteristic curve (AUROC). Calibration was assessed by plotting calibration curves with 95% confidence intervals (CIs) to investigate the relationships between the observed and expected outcomes within each risk decile. Brier's score was also used as an additional parameter for comparing the overall agreement between the predicted and observed outcomes. Funnel plot graphs of SMRs and SRUs considering only those ICUs with greater than 150 admissions were fitted to evaluate potential biases in the estimation of these indicators.

Split sample: the full dataset was temporally stratified into training (1/1/2023 – 3/31/2024) and validation (4/1/2024 – 9/30/2024) samples.

Derivation of the customized version of the SAPS 3 score: After confirming the poor calibration of the original SAPS 3-SE, a first-level customization (recalibration) by computing a new logistic coefficient while maintaining the same variables with the same weights as the original model was performed. A logistic regression was fitted with the SAPS 3 score as the independent variable and in-hospital mortality as the dependent variable in the training dataset (SAPS 3 customized equation, SAPS 3-Custom). The following equation was derived: [-20.9447434 + ln(SAPS 3 score + 1) × 4.894223]. To update the average number of expected ICU days to produce a survivor in our dataset for the purpose of SRU estimation, patients were stratified into the nine SAPS 3 strata (< 24; 25-34; 35-44; 45-54; 55-64; 65-74; 75-84; 85-94; ≥ 95 points) originally proposed by Rothen et al. First, the average number of resources expected to produce a survivor in each stratum was estimated by dividing the sum of the ICU LOS of all patients in that stratum by the number of surviving patients in that stratum. To calculate the SRU for a given ICU, the sum of the ICU LOS of all patients was divided by the sum of the total number of expected days to produce survivors according to the SAPS 3 strata to which patients were assigned. "Zero" expected days was assigned to non-survivors. To avoid noise and the wide variability of the SRUs introduced by patients with disproportionately high ICU LOSs, we truncated it to 30 days (please, refer to e-Table 2 of this ESM).

The statistical analyses were performed using R version 3.5.2 (http://www.r-project.org).

REFERENCES

1. Associação de Medicina Intensiva Brasileira, Epimed Solutions. UTIs Brasileiras. [cited 2025 Oct 2]. Available from: https://www.utisbrasileiras.com/en/
2. Vincent JL, Moreno R, Takala J, Willatts S, De Mendonça A, Bruining H, et al.: The SOFA (Sepsis-related Organ Failure Assessment) score to describe organ dysfunction/failure. On behalf of the Working Group on Sepsis-Related Problems of the European Society of Intensive Care Medicine. Intensive Care Med. 1996;22(7):707-10.
3. Charlson ME, Pompei P, Ales KL, MacKenzie CR. A new method of classifying prognostic comorbidity in longitudinal studies: development and validation. J Chronic Dis. 1987;40(5):373-83.
4. Farhat JS, Velanovich V, Falvo AJ, Horst HM, Swartz A, Patton JH Jr, et al. Are the frail destined to fail? Frailty index as predictor of surgical morbidity and mortality in the elderly. J Trauma Acute Care Surg. 2012;72(6):1526-30; discussion 1530-1.
5. Moreno RP, Metnitz PG, Almeida E, Jordan B, Bauer P, Campos RA, et al.; SAPS 3 Investigators. SAPS 3—From evaluation of the patient to evaluation of the intensive care unit. Part 2: development of a prognostic model for hospital mortality at ICU admission. Intensive Care Med. 2005;31(10):1345-55.
6. Rothen HU, Stricker K, Einfalt J, Bauer P, Metnitz PG, Moreno RP, et al. Variability in outcome and resource use in intensive care units. Intensive Care Med. 2007;33(8):1329-36.
